# Caries Experience Evidenced in Children having Dental Fluorosis

**DOI:** 10.5005/jp-journals-10005-1026

**Published:** 2009-08-26

**Authors:** Tuli A, Rehani U, Aggrawal A

**Affiliations:** 1 Postgraduate Student, Department of Pedodontics, Subharti Dental College, Meerut, Uttar Pradesh, India; 2 Professor and Head, Department of Pedodontics, Subharti Dental College, Meerut, Uttar Pradesh, India; 3 Reader, Department of Pedodontics, Subharti Dental College, Meerut, Uttar Pradesh, India

**Keywords:** Dental fluorosis, Dean’s criteria index, DMFT index.

## Abstract

*Background and objective:* The purpose of this study was to assess the prevalence of caries in children aged 8-13 years having dental fluorosis and to determine the correlation between the grades of dental fluorosis and caries.

*Material and methods:* 451 school children in the age group of 8-13 years were selected for the study and were divided into six age groups. The children were assessed for dental fluorosis according to Dean’s criteria Index of fluorosis, and dental caries according to WHO basic survey guidelines. The overall oral health status of the child was assessed by DMFT index.

*Results:* The results of the present study revealed that the prevalence of grade 2 fluorosis was the highest and grade 5 fluorosis was the lowest in all the age groups. Number of children having dental fluorosis was highest in the age group between 12-13 years followed by the age group between 13-14 years. The overall DMFT increased as the age of the children increased in the different age groups. The DMFT increased as the severity of fluorosis increased upto grade 2 and then decreased from grade 3 to grade 5.

## INTRODUCTION

The discovery of fluoride’s remarkable properties in the prevention of dental caries is a classical example of extensive epidemiological research for the betterment of man. It helped moved dental practice from craft into the science based profession it is today. Earlier it was thought that to exert its maximum cariostatic effect fluoride had to become incorporated into dental enamel during development, and hence it was inevitable to have a certain prevalence of fluorosis in a population. Dental fluorosis was then regarded as an unfortunate side-effect to fluoride’s caries-protective benefits.^1^

Dental fluorosis which results in the hypomineralization of tooth enamel develops due to the continuous ingestion of excessive amount of fluoride during tooth development.^2^ The hypomineralization can be attributed to altered metabolism in any one or all of the phases of amoelogenesis, i.e. altered amoeloblast activity, interference with enamel crystal nuclei, faulty enzymatic relationships, etc.^3^ This results in a variety of pathologic changes in the structure of teeth.^4^ Dental fluorosis if not prevented during childhood can lead to hampered dental esthetics during adulthood. It alters the appearance of the teeth; therefore, the potential consequences of fluorosis are cosmetic. While milder forms of dental fluorosis do not compromise oral health or function, an increase in dental fluorosis may be perceived to affect dental appearance and psychosocial well-being.^4^ Independent to the fluoride concentration in drinking water, caries prevalence has been seen to increase with increasing severity of dental fluorosis in the second molars, first molars, premolars and canines.^5^

Over the last decade there has been some concern that the prevalence of fluorosis has been increasing in a number of countries, India being one of them.^6^ Fluorosis is endemic in 20 states in India on account of excess of fluoride in ground water; therefore, the prevalence of dental fluorosis tends to be a significant problem.^7^ The present descriptive epidemiological study was a conducted in Meerut district to determine the caries experience in children having dental fluorosis and the correlation between these two interrelated oral afflictions.

## MATERIAL AND METHODS

School children in the age group of 8-13 years from the school population of Meerut district and children visiting out patient department (OPD) of Department of Pedodontics and Preventive Dentistry, Subharti Dental College, Meerut were examined. The children were assessed for dental fluorosis and dental caries according to WHO guidelines.^8^,^9^ 451 children having dental fluorosis were selected for the study. Before visiting a school prior permission was taken from the local school authorities,the village pradhan and a public relations officer, who was familiar with the area, informed the local school authorities about the free dental check-up in advance.

The children selected for the study were divided into six groups according to age viz., 8-9 years (Group I), 9-10 years (Group II), 10-11 years (Group III) 11-12 years (Group IV), 12-13 years (Group V) and 13-14 years (Group VI) respectively.

Information was collected via a structured proforma which included demographic variables such as name, age, sex, date of birth, school address along with the scores for dental fluorosis and dental caries. After completing the proforma, intraoral and extraoral photographs of the children in the study were taken to add to the authenticity of the study.

All the data was compiled and subjected to statistical analysis using Unpaired ‘t’test and Karl Pearson correlation coefficient to derive the results. The statistical analysis was done using SPSS/version 11.5 software package. A ‘p’ value of < 0.05 was considered as statistically significant. Single examiner performed all the examination procedure in this study. The single examiner concept maintains consistency and eliminates inter examiner bias.

## RESULTS

**Graph 1:**
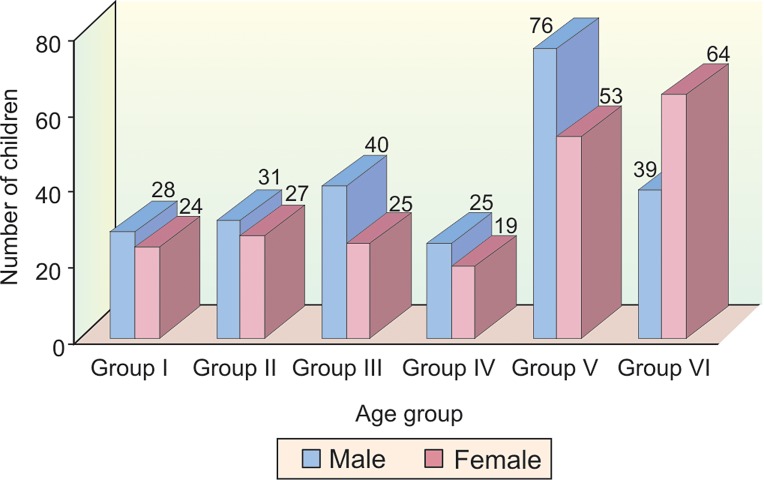
Distribution of sample according to age and gender

The sample which was divided into 6 age groups consisted of 239 (52.99%) males and 212 (47%) females respectively (Table 1 and Graph 1).

**Graph 2:**
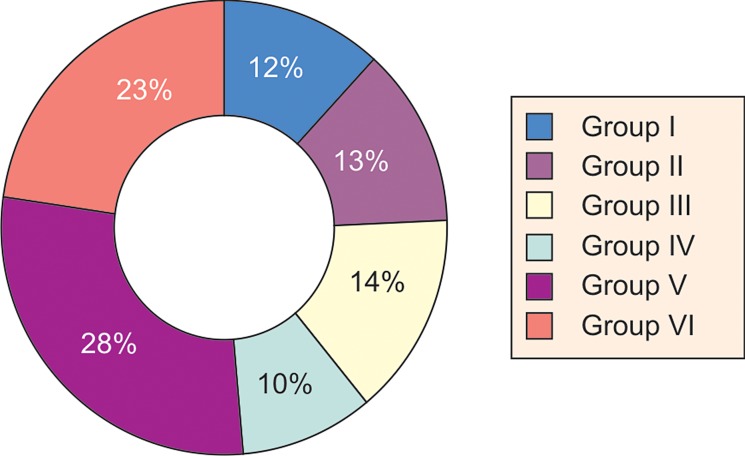
Pie represent showing percentage distribution of sample

The number of subjects belonging to each age group was, group I-52 (12%), group II-58 (13%), group III-65 (14%), group IV-44 (10%), group V-129 (29%) and group VI-103 (23%) (Table 1 and Graph 2).

The number of children having dental fluorosis also varied according to the grades of fluorosis (Table 2).

**Graph 3:**
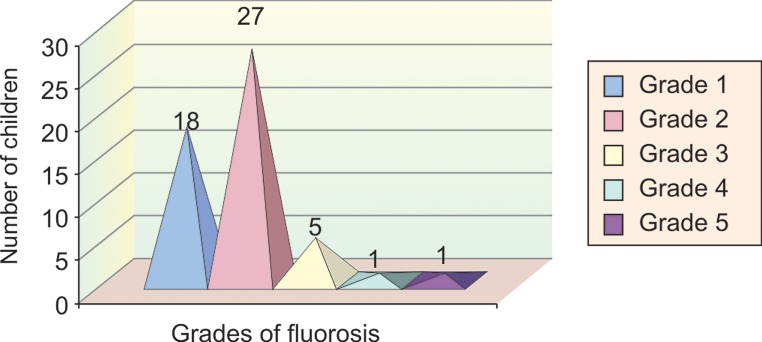
Sample distribution according to grades of fluorosis in group 1

In group I, out of a total of 52 children, 18 children (34.6%) had grade 1 fluorosis, 27 children (52%) had grade 2 fluorosis, 5 children (9.6%) had grade 3 fluorosis, 1 child (1.9%) had grade 4 fluorosis and 1 child (1.9%) had grade 5 fluorosis (Table 2 and Graph 3).

**Table 1:** TABLE 1: Distribution of sample by age and gender

*Age group (Years)*		*Male*				*Female*				*Total*		
		*No.*		*%*		*No.*		*%*		*No.*		*%*
8-9 years (Group I)		28		11.7		24		11.3		52		12
9-10 years (Group II)		31		12.9		27		12.7		58		13
10-11 years (Group III)		40		16.7		25		11.7		65		14
11-12 years (Group IV)		25		10.4		19		8.9		44		10
12-13 years (Group V)		76		31.7		53		25		129		29
13-14 years (Group VI)		39		16.3		64		30.1		103		23
Total		239		52.99		212		47.0		451		100

**Table 2:** TABLE 2: Distribution of sample according to age group, grades and percentage of fluorosis

*Age group *		*No. of children*			*Grade 1*					*Grade 2*					*Grade 3*					*Grade 4*					*Grade 5*		
					*No.*		*%*			*No.*		*%*			*No.*		*%*			*No.*		*%*			*No.*		*%*
Group I		52			18		34.6			27		52			5		9.6			1		1.9			1		1.9
Group II		58			17		29.3			31		53			8		13.7			2		3.4			0		0
Group III		65			20		31.2			25		39			16		25			4		6			0		0
Group IV		44			13		29.5			20		45.4			8		18.1			3		6.8			0		0
Group V		129			23		18			75		58.1			26		20.1			5		3.8			0		0
Group VI		103			22		21.3			49		47.5			27		26.2			5		4.8			0		0
Total		451			113		25			227		50			90		20			20		4.5			1		0.2

**Graph 4:**
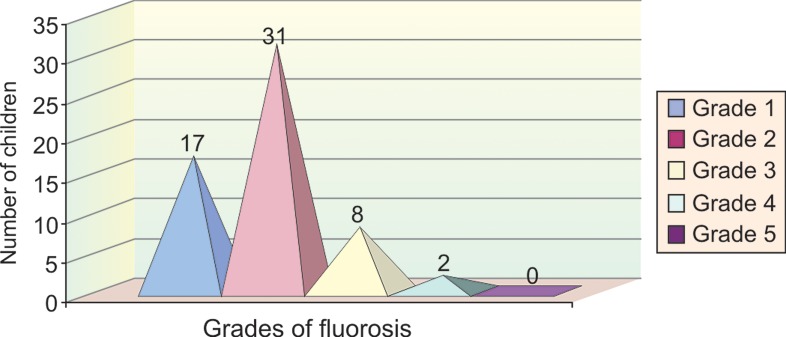
Sample distribution according to fluorosis in group 2

In group II, 17 children (29.3%) had grade 1 fluorosis, 31 children (53%) had grade 2 fluorosis, 8 children (13.7%) had grade 3 fluorosis, 2 children (3.4%) had grade 4 fluorosis and none of the children examined had grade 5 fluorosis (Table 2 and Graph 4).

**Graph 5:**
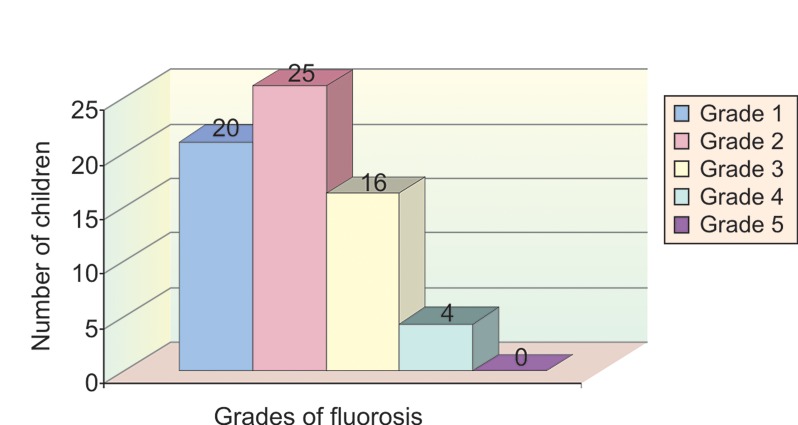
Sample distribution according to grades of fluorosis in group 3

In group III, 20 children (31.2%) had grade 1 fluorosis, 25 children (39%) had grade 2 fluorosis, 16 children (25%) had grade 3 fluorosis, 4 children (6%) had grade 4 fluorosis and none of the children examined had grade 5 fluorosis (Table 2 and Graph 5).

**Graph 6:**
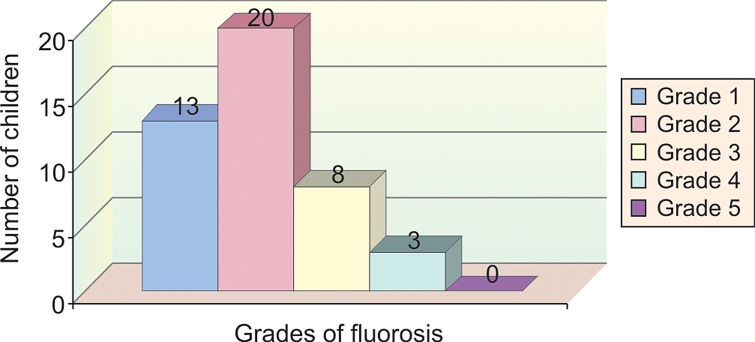
Sample distribution according to grades of fluorosis in group 4

In group IV, 13 children (29.5%) had grade 1 fluorosis, 20 children (45.4%) had grade 2 fluorosis, 8 children (18.1%) had grade 3 fluorosis, 3 children (6.8%) had grade 4 fluorosis and none of the children examined had grade 5 fluorosis (Table 2 and Graph 6).

**Graph 7:**
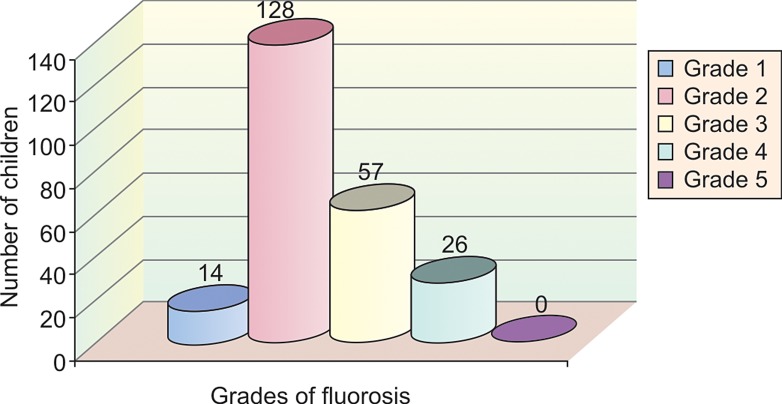
Sample distribution according to grades of fluorosis in group 5

In group V, 23 children (18%) had grade 1 fluorosis, 75 children (58.1%) had grade 2 fluorosis, 26 children (20.1%) had grade 3 fluorosis, 5 children (3.8%) had grade 4 fluorosis and none of the children examined had grade 5 fluorosis (Table 2 and Graph 7).

**Graph 8:**
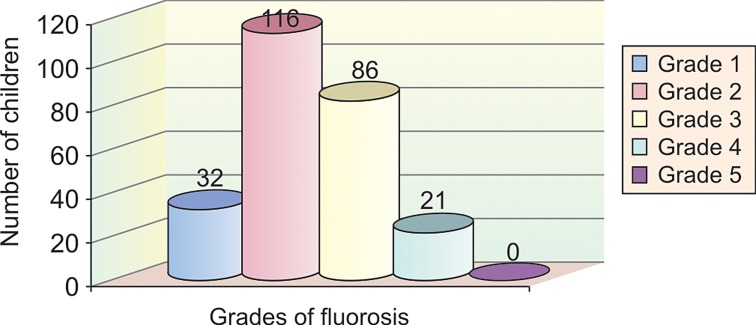
Sample distribution according to grades of fluorosis in group 6

In group VI, 22 children (21.3%) had grade 1 fluorosis, 49 children (47.5%) had grade 2 fluorosis, 27 children (26.2%) had grade 3 fluorosis, 5 children (4.8%) had grade 4 fluorosis and none of the children examined had grade 5 fluorosis (Table 2 and Graph 8).

In the present study it was observed that in all the age groups as the severity of fluorosis increased from grade 1 to grade 2, the number of children in each age group increased but later decreased, i.e. the number of children having grade 5 fluorosis were the least.

**Graph 9:**
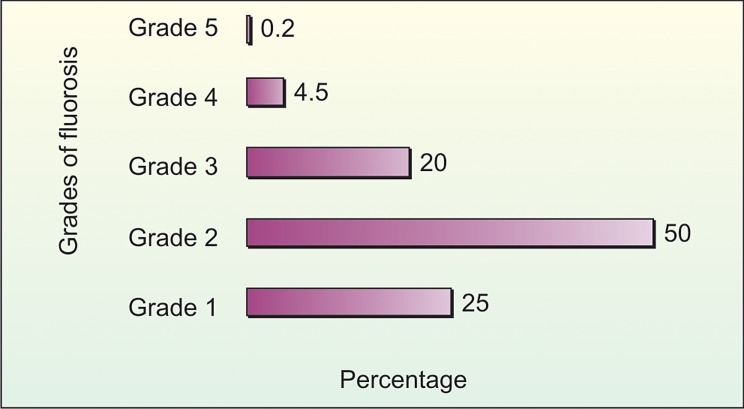
Percentage distribution of fluorosis according to grades

Also it was noted that the percentage of fluorosis increased from grade 1 to grade 2 and then decreased from grade 3 to grade 5. The overall percentage of grade 1 fluorosis was 25%, grade 2 fluorosis was 50%, grade 3 fluorosis was 20%, grade 4 fluorosis was 4.5% and grade 5 fluorosis was 0.2% (Table 2 and Graph 9).

At different grades of dental fluorosis the DMFT recorded also varied. The overall DMFT increased as age group increased. The DMFT increased from group I (59) to group II (89), then decreased from group III (85) to group IV (78) and then increased from group V (225) to group VI (255). The highest DMFT was recorded in group VI (255) followed by group V (225) (Table 3).

The mean DMFT also varied in different age groups according to the grades of fluorosis (Table 3).

In group III it was noted that as the severity of fluorosis increased the DMFT increased from grade 1 to grade 2 and remained static at grade 3 and then decreased from grade 4 to grade 5.

In groups I,II,V and VI it was noted that as the severity of fluorosis increased from grade 1 to grade 2 the DMFT increased and then decreased from grade 3 to grade 5, i.e. the number of children having DMFT in grade 5 fluorosis were the least.

The mean DMFT decreased from group I (1.74 ± 1.31) and group II (1.62 ± 1.04) to group III (1.38 ± 1.16) and then increased from group IV (1.59 ± 0.80) and group V (1.93 ± 1.96) to group VI (2.23 ± 1.17).

**Table 3:** TABLE 3: Mean DMFT according to age group and grades of fluorosis

*Grades of fluorosis*	*Group I*						*Group II*						*Group III*						*Group IV*						*Group V*						*Group VI*					
	*No.*		*DMFT*		*Mean*		*No.*		*DMFT*		*Mean*		*No.*		*DMFT*		*Mean*		*No.*		*DMFT*		*Mean*		*No.*		*DMFT*		*Mean*		*No.*		*DMFT*		*Mean*	
Grade 1	18		15		0.83		17		19		1.11		20		10		0.5		13		13		1		23		14		0.6		23		32		1.45	
Grade 2	27		30		1.11		31		47		1.11		25		31		1.24		20		35		1.75		75		128		1.70		49		116		2.36	
Grade 3	5		9		1.8		8		16		2		16		31		1.93		8		23		2.87		26		57		2.19		27		86		3.18	
Grade 4	1		4		4		2		7		3.5		4		13		3.25		3		7		2.33		5		26		5.2		5		21		4.2	
Grade 5	1		1		1		0		0		0		0		0		0		0		0		0		0		0		0		0		0		0	
Total	52		59		8.74		58		89		8.12		65		85		6.92		44		78		7.95		129		225		9.69		103		255		11.19	
Mean	1.74 ± 1.31						1.62 ± 1.04						1.38 ± 1.16						1.59 ± 0.80						1.93 ± 1.96						2.23 ± 1.17					
± **SD**																																				

**Table 4:** TABLE 4: Correlation coefficient between grades of fluorosis and DMFT for different age groups

*Age group*		*Group I*		*Group II*		*Group III*		*Group IV*		*Group V*		*Group VI*
**r**		–0.74440*		–0.6865		–0.4400		0.6214		–0.4035^#^		–0.5211
r = Correlation coefficient												
* = Strong negative correlation												
# = Weak negative correlation												

**Table 5:** TABLE 5: Significance of correlation between grades of fluorosis and DMFT for different age groups

*S. no*		*Age group*		*t cal*		*t tab*		*P value*
1		Group I (8-9 years)		–23.5931**				P << 0.05
								P << 0.05
2		Group II (9-10 years)		–19.29		t (0.05) = 1.962		Significant
3		Group III (10-11 years)		–10.38		t (0.01) = 2.58		Significant
4		Group IV (11-12 years)		–16.8053				Significant
5		Group V (12-13 years)		–9.3444*				P << 0.05
								P << 0.05
6		Group VI (13-14 years)		–12.9369				Significant
** = Highly significant, * = Significant, at 5% and 1% level of significance								

It was found that the correlation between fluorosis and DMFT was significant for all the age groups. There was a strong negative correlation between increasing grades of fluorosis and DMFT in group I (8-9 years). The correlation between fluorosis and DMFT was highly significant for group I (8-9 years). There was a weak negative correlation between increasing grades of fluorosis and DMFT in group V (12-13 years). The correlation between fluorosis and DMFT was least significant for group V (12-13 years) (Tables 4 and 5).

## DISCUSSION

70-80% of districts in Uttar Pradesh are affected by fluorosis, Meerut being one of the fluoride endemic areas in Uttar Pradesh.^10^ A number of studies have been conducted in various parts of India to collect epidemiological data on fluorosis and dental caries but still current update is required. This was one of the main reasons for selecting this area for the present investigation.

Various studies^11^,^12^ conducted to assess dental fluorosis have reported a high prevalence of grade 2 fluorosis in school children. Our study is in accordance with them as the percentage of grade 2 fluorosis is highest in our study. The reason for this observation could be that the fluoride levels in the water supply may be low, so as to cause only grade 2 fluorosis. Even if the fluoride levels are high in the water supply, as the climatic and lifestyle patterns differ at different geographical locations, the effect of fluorides also differ; being more pronounced in drier, hotter more arid regions where there is increased intake of water.

The present study observed that the highest number of children having dental fluorosis was in the age group of 12- 13 years which coincided with the findings reported by Baelum V, Manji F, Fejerskov O.^13^ It may indicate that several years after tooth eruption, there was a trend towards an increasing enamel surface destruction in children exhibiting pronounced degrees of subsurface enamel hypomineralization at the time of eruption.

J Mann, M Tibi, HD, S Cohen^14^ reported that the overall percentage of dental fluorosis was more in males than in females which was also observed in our study. This could be attributed to the increased number of males than females in a particular area.

The number of children having grade 5 fluorosis was the least in our study. This is in accordance with the findings reported by Menon A, Indushekhar KR.^11^

The results of the present study conducted also revealed that the overall DMFT increased as the age of children increased in the various age groups which coincided with the studies conducted by M Kumar P, Joseph T, Varma RB, Jayanthi M.^15^ Age may act as a confounding factor as far as the relationship between caries and age is observed. The reason may be that as the age of the children increases they experience changing lifestyles and increased exposure to cariogenic diet from the time of tooth eruption till the time teeth are *in situ*.

Authors^16^ have reported as the severity of dental fluorosis increased the DMFT increased upto the level of mild fluorosis and then decreased as the severity increased from moderate to severe fluorosis. These findings were similar to the results found in our study.

It was observed that the correlation between fluorosis and DMFT was significant for all the age groups, as was also reported by YE Ibrahim, K Bjorvatan and JM Birkeland.^17^ The increasing severity of fluorosis which may lead to enamel defects may be responsible for variation in the DMFT and significant correlation between the two factors.

It was also observed that the correlation between fluorosis and DMFT was highly significant for group I (8-9 years). There was a strong negative correlation between increasing grades of fluorosis and DMFT in group I (8-9 years). This may be attributed to the factors such as less number of permanent teeth, early initiation of proper oral hygiene measures as well as early detection of carious lesions.

The data reported by Acharya S, Anuradha KP^16^ found a negative correlation between DMFT and increasing level of fluoride in water in 12-15 years old school children which was in conformity with the results of our study. The reason for the present observation could be that as the age of the children increases, they are exposed to changing patterns of dietary intake, oral hygiene methods, variable geographic locations, therefore, fluorosis cannot be judged as the single factor responsible for increasing or decreasing the DMFT.

## CONCLUSION

Epidemiological surveys in other areas of Meerut district would further enhance our knowledge on the prevalence of dental fluorosis, prevalence of DMFT and correlation between these two prevalent oral afflictions. From this study we can conclude that dental fluorosis is present in Meerut district and dental caries was seen in conjunction with fluorosis in all the age groups of children but the correlation between these two variables was the strongest in the lower age groups.

The patients with severe fluorosis should be followed up by epidemiological surveillance, in order to control the occurrence of new cases. It is important to have rigorous control of oral health educative and preventive programes on dental fluorosis and its effects on oral health. Current knowledge of all oral diseases affecting children and application of recent scientific advancements in prevention or further spread of the oral afflictions is the need of the hour. Since children and adolescents are most commonly affected by fluorosis and dental caries therefore, constant update of the prevalence of caries and fluorosis with a larger sample is required and preventive programes need to be focused on them.
